# Dispersed white roots in red beetroot influence the accuracy of root identification based on colours for intercropping studies

**DOI:** 10.1186/s12870-023-04414-5

**Published:** 2023-09-09

**Authors:** Yue Xie, Sindhuja Shanmugam, Hanne Lakkenborg Kristensen

**Affiliations:** 1 Department of Food Science, Aarhus University, Agro Food Park 48, 8200 Aarhus N, Denmark; 2 Department of Vegetables, College of Horticulture, China Agricultural University, Beijing, 100193 China

**Keywords:** Betacyanin, Multispecies, Root study methods, Root proliferation, Root age

## Abstract

**Purpose:**

Beetroot is a model crop for studying root competition in intercropping systems because its red-coloured roots facilitate non-destructive visual discrimination with other root systems of intercropped plants. However, beetroot also has white roots, which could alter how root competition is interpreted. Here we investigated the quantity of white versus red roots in beetroot to quantify the effect of this phenomenon.

**Methods:**

Beetroot was mono-cropped or inter-cropped with white cabbage in a field trial. The distribution of beetroot roots was recorded to 2.5 m soil depth on three dates following the minirhizotron method. Roots in each 0.5 m soil layer were counted and categorised into groups based on colour (white roots, coloured roots, and white roots traced back to be coloured) to investigate the influence of white roots on accuracy of root registration. A pot experiment was conducted with three cultivars to verify if white roots are a general characteristic of beetroot.

**Results:**

White roots in mono-cropped beetroot represented 2.5–4.8% of total roots, on average, across the rooted soil profile. However, white roots represented 6.9% and 11.6% of total roots in the deepest soil layer during August and October, respectively. White roots caused mono-cropped beetroot roots to be underestimated by 1–22% based on root colour discrimination. However, tracing white roots backwards and forwards to coloured parts of roots reduced underestimates to 0.5–15%. Intercropping did not influence the traceability of white roots compared to monocropping. The highest occurrence of white roots appeared during the early growth period and in the deepest soil layers, indicating a linkage to younger roots or higher root proliferation rates.

**Conclusion:**

Beetroot represents a model crop for visual studies linking eco-physiology and root proliferation. The white roots of beetroot must be incorporated by studies of root competition in intercropping systems that use colour as a criterion.

**Supplementary Information:**

The online version contains supplementary material available at 10.1186/s12870-023-04414-5.

## Background

Exploring belowground inter-specific interactions provides important information on the yield and enhancement of nutrient use in intercropping systems, where two or more crops share an overlapping growing period and space. Root systems are primarily studied by collecting root fragments in certain soil volumes, or by visually measuring root development and distribution from images of certain soil interfaces [16]. The latter is non-destructive and less labour-intensive. It allows continuous observations of root systems at certain time intervals and could potentially reveal the dynamics of root growth and traits over time [28]. Visual measurements are commonly conducted using rhizotrons and minirhizotrons in both field and controlled environment (e.g., greenhouse) experiments.

However, discriminating the root systems of intercropped species is challenging when using direct visual measurements, due to the lack of distinct morphological or colour differences in roots. Methods for root discrimination developed based on anatomical, morphological, biochemical, and genomic properties of roots are currently only applicable to destructive methods [17]. Applications of other methods, such as staining roots with colourants and using labelling or natural abundance of isotopes, are limited to plant size, specific plant taxa and environments [4, 7]. Beetroot (*Beta vulgaris* L.) has naturally pink to red-coloured roots, due to the presence of the water-soluble pigment, betacyanin, which is stored in vacuoles [1]. The distinct colour of beetroot roots allows their root systems to be discriminated from companion species in intercropping systems and has been used in combination with the non-destructive methods, rhizotron and minirhizotron [2, 25].

However, to date, all studies using beetroot have assumed that all beetroot roots are pink, red or purple. Yet, bright and shiny white root ends of beetroot (cultivar Edmand's Blood Turnip) were first reported almost 100 years ago, in an early detailed study on root growth by the excavation method [26]. In our recent field experiments using minirhizotrons [10, 21], we confirmed the existence of white-coloured roots of beetroot (cultivars Wodan and Forono). The beetroot root system develops by extending both vertically and horizontally over time. The taproot grows vertically downwards, and the lateral roots grow horizontally when close to the taproot, then turn abruptly downwards [26]. The growing pattern of the beetroot root systems means that the average root age decreases with soil depth. The reported white root ends of beetroot [26] indicate that white-coloured roots are exclusive to young roots. This assumption is supported by the fact that betacyanin is stored in vacuoles, which become central vacuoles in mature root cells. However, we found that the white colour was not exclusive to root ends. To the best of our knowledge, the dispersed white colour in beetroot root systems has not been reported previously, and the occurrence has not been quantified. Yet, it is important to document this information, particularly when using beetroot to study root competition in intercropping systems.

Root systems exhibit plasticity, enabling crops to adapt to various environments. For instance, the morphology of crop root systems responds to soil nutrient concentrations and spatial heterogeneity [11]. In intercropping systems, the morphological traits of root systems might be modified by recognizing neighbouring, genetically different, crops [20]. Root morphology is mainly modified by changes in the spatial root distribution, root length density, root diameter, and proliferation of fine roots and root hairs [12]. However, no studies have detected or reported the effect of intercropping on root colour, related to the modification of edaphic conditions by intercropping.

In addition, the existence of white roots could affect the root counting/registration of each species in rhizotron/minirhizotron methods when the criterion for root discrimination is based on the colour of beetroot roots. Therefore, here, we investigated the distribution of white colour in beetroot roots under field conditions, and to what extent white roots impact the accuracy of root discrimination when using different criteria based on root colour. We hypothesized that: 1) white roots would occur in the fully rooted soil profile of beetroot; 2) abundant white roots would result in a significant underestimate of beetroot root density, while tracing roots backwards and forwards can decrease the underestimate; 3) abundance of white roots would be correlated in time and space with the rate of root proliferation (e.g. more white roots would be found at greater soil depths due to a higher root proliferation); thus, the abundance of white roots would decrease with root age; and 4) intercropping will decrease traceability of white roots due to interspecific interactions compared to monocropping. A supplementary pot experiment was conducted to verify if white roots are a general characteristic of beetroot among cultivars.

## Methods

### Field experiment setup and management

A field study was conducted in an organically managed field at the experimental site AU-Årslev, Denmark (10° 27'E, 55° 18'N) in 2019 to investigate the distribution of white colour in beetroot roots under monocropping and intercropping systems. Originally, the experiment had two factors: cropping system and fertiliser type, with a completely randomised block design including three replicates. A deep red-coloured beetroot (cultivar Forono, Bingenheimer Saatgut) was grown alone (monocrop) or with white cabbage (*Brassica oleracea* L. var. *capitata* f. *alba*, cultivar Storage no. 4) (intercrop). Organic fertiliser treatments of green manure or animal slurry, were applied in May 2019, before sowing or planting aimed at a rate of 160 kg N ha^−1^ of plant available N, taking soil mineral N, soil potential N mineralisation, winter catch crop N, and fertiliser N into account. Shanmugam et al. [21] reported there was no fertiliser effects on root growth of beetroot in 2019. Therefore, we combined two fertiliser treatments into one in this study, leading to a new experimental setup with one factor (cropping systems) and six replicates. Each replicate had nine rows of crops with a row distance of 0.5 m. The soil type of the experimental field was sandy loam (Typic Agrudalf). The soil texture and chemical properties were 13% clay, 15% silt, and 70% sand in the 0–0.25 m soil layer; 15% clay, 15% silt, and 69% sand in the 0.25–0.5 m soil layer; 19% clay, 13% silt, and 68% sand in the 0.5–1 m soil layer; 18% clay, 13% silt, and 68% sand in the 1–2.5 m soil layer. The top 0.25 m soil contained 1.9% organic matter, 1.3 g kg^−1^ total N, 30 mg kg^−1^ P, and 155 mg kg^−1^ K, with a soil pH_CaCl2_ of 6.3 [21]. The interrow plant distances were 0.04 m for sowed beetroot and 0.35 m for transplanted white cabbage. In the intercropping system, every second row of beetroot was replaced with white cabbage. The crops grown in the year previous to the experiment (2018) were the same; however, beetroot rows in 2018 were in the position of the white cabbage rows in 2019, and vice versa. Beetroots were sown on 6 June 2019, and 28-day-old cabbage seedlings were transplanted on 25 June 2019. Irrigations were sprinkled throughout the growing season. Further details on field experimental design, crop rotation, irrigation, fertilisation, and other management operations of this field experiment are provided by Shanmugam et al. [21]. Tillage, winter catch crops, irrigation, and fertilisation were implemented in the same way in mono- and intercropping systems. Pre-crops for beetroot in 2019 were white cabbage in 2018 followed by winter catch crops in both cropping systems.

### Root recording

The distribution of roots beneath the beetroot rows in both monocropping and intercropping systems was recorded using the minirhizotron method with 3-m-long minirhizotrons. We did not include observations of roots in white cabbage rows in intercropping system, because very few red roots were observed. Two 40 mm × 40 mm counting grids were symmetrically painted on the left and right sides of the upper surface of minirhizotron tubes. Each minirhizotron tube was installed at a 30° angle from vertical underneath one beetroot row in each plot (guard rows in each plot were excluded) after sowing, leading to 6 replicates for both monocropping and intercropping systems. Roots were filmed three times during the growing season with a mini video camera (resolution, 800 × 600 pixels); on 26 July, 24 August, and 22 October 2019.

### Root category and registration

To investigate the accuracy of root discrimination under different criteria based on root colour, we categorised beetroot roots into different groups based on root colours. We initially recorded total roots (T) and categorised roots visually based on whether they were coloured (visually pink, red, or purple hue, VC) or white (visually white hue, VW) in both cropping systems. During this process, we observed that a visually white root in an observation window could be traced backwards or forwards (e.g., a few centimetres), along the root and identified as having pink or red colour outside the specific grid window. That is, the root was coloured in a length fraction that was positioned outside of any window, inside a previous or later window or attached to coloured roots with a topologically higher or lower order (Figure S2). Thus, we registered these roots as traceable white roots to coloured roots (WC) in both cropping systems. Eventually, the coloured roots (C) category consisted of the sum of visually coloured roots (VC) and WC, whereas white roots (W) were visually white roots with WC excluded in this study (Table 1). The root categories are unique to this study compared to any previous studies e.g., that of Shanmugam et al. [21], where roots were counted irrespective of colour.

**Table 1:** Root colour categories and corresponding root registration for beetroot in the field study

Root category	Definition	Criterion for identifying beetroot roots
Visually coloured root (VC)	Roots visually have pink, red or purple hue	Visually coloured root in the observed window
Visually white root (VW)	Roots visually have white hue	
Traceable white root to coloured root (WC)	White root in the observed window could be traced backward or forward to coloured roots	
Coloured root (C)	VC + WC	Visually coloured root Traceable white root traced back as coloured root
White root (W)	VW-WC	
Total roots (T)	All roots presented in the window	All roots presented in the observed window

Root intersections were manually counted as the total number of differently categorised roots crossing the grid lines in each 40 mm × 40 mm counting grids [10], based on the line intersect method [23]. Root counts were summed for each 0.5 m soil depth interval.

### Estimating beetroot roots under different criteria

Because white cabbage roots (in white) were present in the intercropping system, it was not possible to identify and register the white roots of beetroot in the intercropping system. Therefore, we only investigated how abundance of white roots would influence the estimation of beetroot root density in monocropping systems. In the monocropping system, VC, C, and T counts additionally represented three criteria for identifying the roots of beetroot. VC corresponded to the criterion that a beetroot root is only registered when it has coloured roots in the observed window. C corresponded to the criterion that a beetroot root was registered when it was coloured in the window (VC) or when a white root could be traced back to a coloured root (WC). T corresponded to the actual root count of beetroot, which represented 100% of beetroot roots (Table 1).

### Pot trial setup and management

A pot trial was conducted to validate our field results. In the pot trial, we documented the occurrence of white roots in three different beetroot cultivars. Three deep red-coloured beetroot cultivars were selected: Forono (Bingenheimer Saatgut), Kogel-2 (Solsikken), and Cylindra (Solsikken). These cultivars were grown in pots (3.5 l; 17 cm in height and 16.5 cm in diameter) in a greenhouse at the Department of Food Science, Aarhus, Denmark. A 4 cm × 4 cm window was cut on the side of each pot, 5 cm from the bottom. A transplant plastic sheet was used to cover the window from the inside to observe roots. Light-tight material was used to cover the window (and block out light) from the outside during the trial. Ten seeds of the same cultivar were sown in each pot, which was packed with sandy soil mixed with organic fertiliser (N-P-K: 2–1-2) on 31 January 2022. The experiment was a completely randomised design with three replicates. Irrigation was given in the form of tap water. To avoid water stress on plants, the water status of soil surface was checked daily by experienced gardeners. At dry soil surface, irrigation was applied by via rubber pipe until soil water content reached field capacity. The temperature was set at 22 °C. Ventilation was started when the temperature exceeded 24 °C. The photoperiod was set to a 16 h day^−1^. Additional light of 250 mmol light emitting diode (LED) light (FL300 Growth, Senmatic, Sonderso, Denmark) was given when natural light was below 200 mmol.

### Root measurements

Roots were measured 28 days after beetroot was sown (28 February 2022), following the methods of Čereković et al. [5]. At the end of the pot trial (28 February 2022), beetroot roots were sampled by destructive methods. Entire beetroot plants were first taken out of the pots carefully. Then, all lateral roots were collected by severing the lateral roots from the tap roots and manually extracting most roots from the soil. Soil attached to the collected roots was removed by rinsing roots with tap water in sieves (mesh size 2 mm). A weight-based subsample was assimilated by separating out one-tenth of each collected root sample. Subsamples were displayed in tap water in transplant trays. The roots were categorised into white, red, and other (mainly yellow and brown) coloured roots. The proportions of the three colour categories to total roots were evaluated visually by 10 randomly selected people on code-marked samples at different times.

### Data analysis

We assessed how white roots and traceable white roots affected the registration of beetroot roots using data from the monocropping system as a proxy, due to the unavailability of white roots of beetroot in the intercropping system. It was not possible to age roots through direct visual observations. Instead, we assessed the spatiotemporal distribution of colour in the root systems (5 soil layers across three dates) as a proxy for assessing the effects of root age on root colour.

To quantify how white roots and traceable white roots affect the registration of beetroot roots, root registration under different criteria was compared, by using the proportions of registered root counts to total beetroot root count. The effects of date and soil layer on W counts and their proportions to T counts were assessed in the monocropping system. The effect of the intercropping system on WC counts and their proportions to C counts was assessed by comparing root counts and proportions between the two cropping systems.

R software (version 4.1.2) was used for the statistical analysis. Root count data were analysed using General Linear Model defined according to the Poisson distribution. When data were under-dispersed or over-dispersed, quasi-Poisson or negative binominal distribution was defined in the models. The proportions of W, C, and WC counts to T counts, and the proportion of WC counts to C counts, were analysed with Linear Models when homogeneity of data was obtained by transforming data with functions of y = x^1/2^ or y = log(x).

## Results

### Distribution and abundance of white roots in the monocropping system

**Fig. 1:**
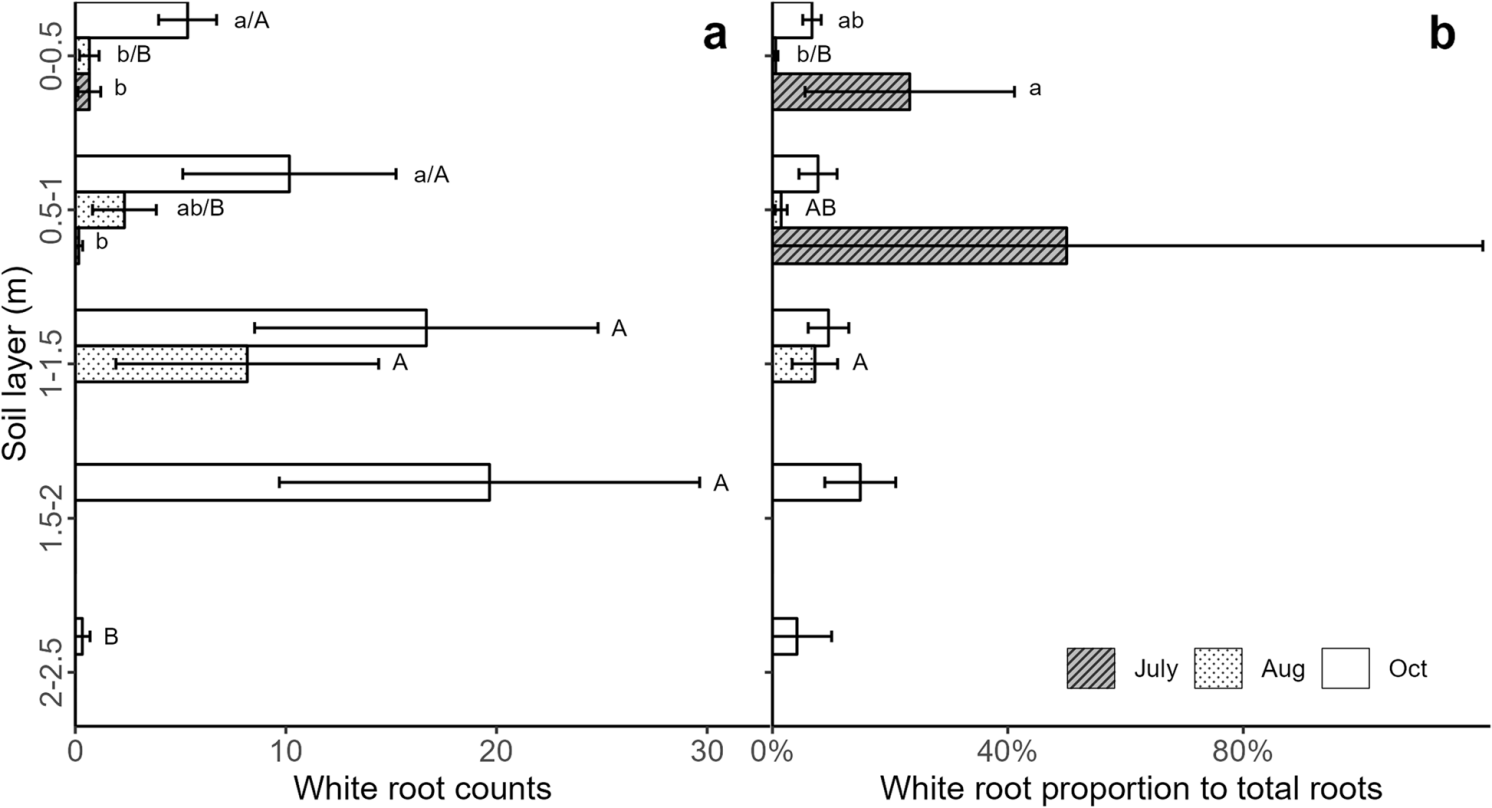
The count of white roots (W) (**a**) and the proportion of white root count to the total root count (T) (**b**) in each soil layer and at each date, in the monocropping system of beetroot. The lowercase letters indicate the significant difference among dates in each soil layer at *p* < 0.05 (*n* = 6). The uppercase letters indicate the significant difference among soil layers at each date at *p* < 0.05 (*n* = 6)

The white colour was observed at any depth or part of the roots (Figure S1a and b). In the field trial, the abundance of white beetroot roots (W), on average, across the rooted soil profile was 3.3% in July, 2.5% in August, and 4.8% in October. The abundance of W increased over time in the 0 − 0.5 m and 0.5 − 1 m soil layers, with higher root counts in October compared to July (both soil layers) and August (0.5 − 1 m) in the monocropping system (Fig. 1a). In August, roots were only distributed in the 0 − 1.5 m soil layer. White root count was higher in the 1 − 1.5 m soil layer compared to the 0 − 0.5 m and 0.5 − 1 m soil layers (*p* < 0.05). In October, the lowest W count was recorded in the 2 − 2.5 m soil layer (Fig. 1a). The proportion of W to T counts was higher in July compared to August (*p* < 0.05) (Fig. 1b). In August, the proportion of W to T counts was 6.9% in the 1 − 1.5 m soil layer and was higher compared to the 0 − 0.5 m soil layer (*p* < 0.05). In October, the proportion of W to T counts was 11.6% in the 2 − 2.5 m soil layer and was similar in all soil layers (Fig. 1b).

Similarly, white roots were recorded in the cut windows of pots in the pot trial (Figure S2c, d and e). The proportions of white roots (W) to T counts in three cultivars, Forono, Kogel-2, and Cylindra, in the pot trial were 13%, 24%, and 3%, respectively, at the end of the experiment (Table S1).

### Counts of beetroot roots under the three criteria in the monocropping system

**Fig. 2:**
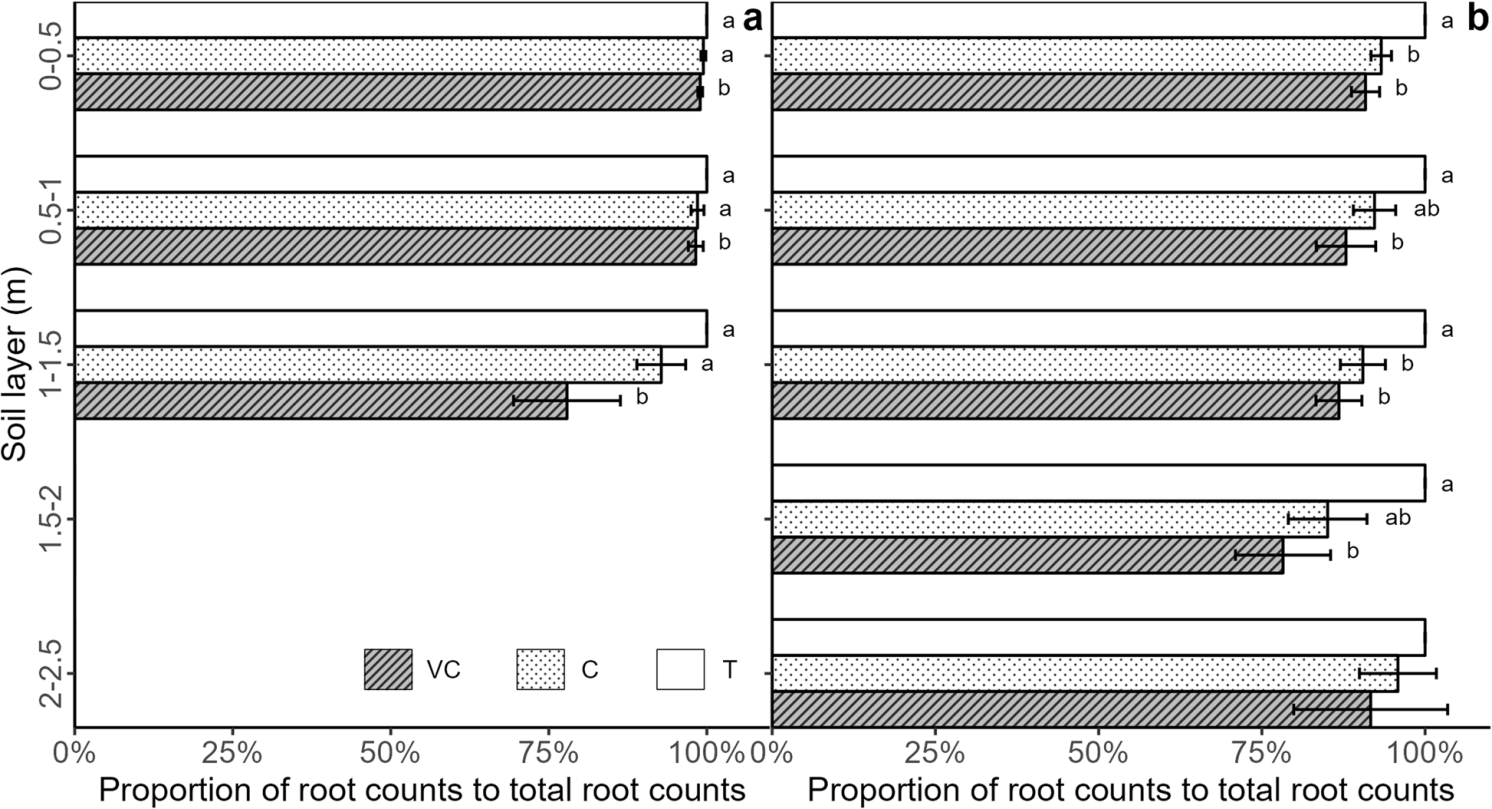
The proportions of visually coloured (VC) and total coloured (C) root count relative to total root count (T) set at 100% in mono-cropped beetroot based on three criteria in August (**a**) and October (**b**). The lowercase letters indicate the significant difference among criteria in each soil layer at *p* < 0.05  (*n* = 6)

White colour in beetroot roots began affecting the root registration of mono-cropped beetroot in August and October (Fig. 2). Root registration in each soil layer was lowest under the VC criterion compared to the other two criteria in both months. In August, root registration under the VC criterion was 99%, 98%, and 78% of T counts in the 0 − 0.5 m, 0.5 − 1 m, and 1 − 1.5 m soil layers, respectively. This resulted in a 1% and 22% underestimation of coloured roots, which was significantly lower compared to the T criterion (*p* < 0.05). Root registration in the 1 − 1.5 m soil layer tended to be significantly lower under the C criterion compared to the T criterion (*p* = 0.0677), accounting for 93% of T count (Fig. 2a). Under the C criterion, counts were underestimated by 0.5%, 1.5%, and 7% in the 0 − 0.5 m, 0.5 − 1 m, and 1 − 1.5 m soil layers, respectively. In October, root registration was lower under the VC criterion compared to the T criterion in the 0 − 0.5 m, 0.5 − 1 m, 1 − 1.5 m, and 1.5 − 2 m soil layers (*p* < 0.05), with estimates of 91%, 88%, 87%, and 78% of T counts, respectively. This resulted in underestimates of 8% to 22% (Fig. 2b). Root registration under the C criterion was 93% and 90% of T counts in the 0 − 0.5 m and 1 − 1.5 m soil layers, respectively (*p* < 0.05) (Fig. 2b). Thus, root counts were underestimated under the C criterion by 6.7%, 7.7%, 9.5%, 14.9%, and 4.2% in the 0 − 0.5 m, 0.5 − 1 m, 1 − 1.5 m, 1.5 − 2 m, and 2 − 2.5 m soil layers, respectively. No significant difference was detected in root registration under VC and C criteria in any of the soil layers.

### Traceable white roots in the two cropping systems

**Fig. 3:**
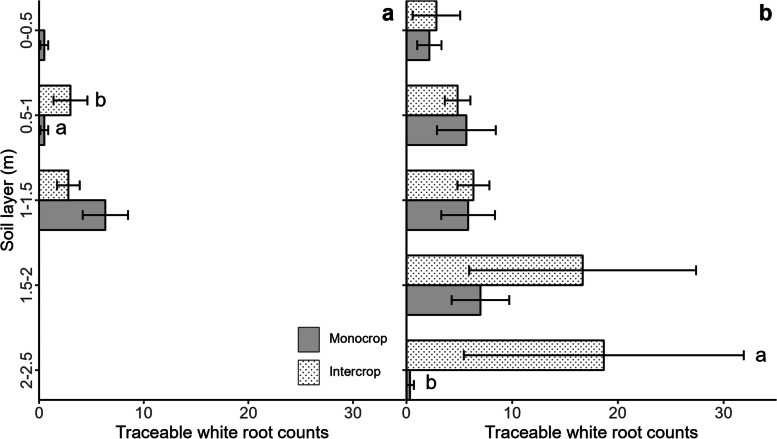
The traceable root count (WC) in each soil layer in August (**a**) and October (**b**), in monocropping systems of beetroot and intercropping systems of beetroot and cabbage in minirhizotrons placed in beetroot rows. The lowercase letters indicate the significant difference between cropping systems in each soil layer at *p* < 0.05 (*n* = 6)

Differences in WC counts appeared in specific soil layers in August and October. In August, WC count in the 0.5 − 1 m soil layer was higher in the intercropping system of beetroot and cabbage compared to the monocropping system of beetroot (Fig. 3a). In October, WC count in the 2 − 2.5 m soil layer was higher in the intercropping system compared to the monocropping system (Fig. 3b). However, the proportions of WC root counts to C root counts were similar (range: 0–19%) in each soil layer of the two cropping systems at each date (results not shown).

## Discussion

### Abundance of white-coloured beetroot roots

White roots, which could not be traced back to red-coloured roots, were found in all soil layers of the rooted soil profile of beetroot. This finding confirmed our first hypothesis that white roots occur in the fully rooted soil profile of beetroot and supports the early study of Weaver and Bruner [26]. White-coloured roots exhibited a clear spatiotemporal pattern in development. First, the summed W counts in the whole soil profile increased from 2 to 104 from July to October. Second, W counts were higher in the deeper soil layers in August (1 − 1.5 m) and October (1.5 − 2 m) (Fig. 1a). This spatiotemporal pattern reflected the development of the beetroot root system, which extends both horizontally and vertically in the soil over time [13, 26]. Although the proportions of W counts to T counts were only 2.5–4.8%, on average, across the fully rooted soil profile (data not shown), there was distinct variation across soil layers. For instance, the proportion of W count was 6.9% (August) and 11.6% (October) in the deeper soil layers (1 − 1.5 m and 1.5 − 2 m, respectively), but barely any white roots were detected in some other soil layers (Fig. 1b). The low W count and proportion in 2 − 2.5 m soil layer in October could be due to reduced investment in new roots in deep soil layers in late season, which is a common strategy in summer crops [13] (Fig. 1), in contrast to autumn catch crops [28]. The occurrence of white roots in all three beetroot cultivars in the pot trial (Table S1) supported our field results (including Forono, which was used in both the pot and field trial). The occurrence also supported our hypothesis that white roots are a general characteristic of beetroot root systems at least at early growing stage (28 days), although the abundance of white roots did differ between cultivars. The root colour of beetroot is determined by the contents of betalain, whose biosynthesis has been proved to be a synthetic consequence of genetics and abiotic conditions [3, 22]. Root colour has been used as an indicator of root age in many studies, where the root colour changes are linked to root senescence [18]. However, the formation of coloured roots of beetroot is under a completely different mechanism. Further studies should be conducted with a longer period to confirm the link between root age and colour changes in beetroot roots of different cultivars. If the link is confirmed, the white root abundance might change for full grown beetroot cultivars. The white roots in full grown crops of Forono in the field trial can be due to high root branching patterns and root turnover rates. More white roots can be expected in cultivars with high root branching and turnover rates, which may lead to more root tips and new roots, respectively.

### High occurrence of white roots links to younger roots

The white roots traced back to red-coloured roots in the upper soil layers of the field indicated that white-coloured roots were not exclusive to root tips in the present study (Figure S3). This result contrasted with the conclusion of Weaver and Bruner [26]. However, white roots were strongly associated with roots of younger age, on average. The higher proportion of white roots to total root counts in July (in the top 0.25 m soil layer) and in the deeper soil layer (1 − 1.5 m, in August) indicated that more white roots were present at certain time points and in spaces with higher root proliferation rate and vigorous roots. This result confirmed our third hypothesis that the red colour of beetroot roots develops with increasing root age. The occurrence of white root tips, as reported by Weaver and Bruner [26], might be attributed to the lack of central vacuoles, which form in mature cells, with vacuoles being the organelles for betacyanin storage. The existence of dispersed white colours in red beetroot roots has not previously been reported, and its cause remains unclear. The metabolism of betalains (red betacyanins and yellow betaxanthins could be stimulated by both abiotic and biotic stress [6, 24]. However, the white colour of beetroot roots in our case could not be primarily linked to environmental stresses, because the occurrence of white roots exhibited a spatiotemporal pattern. In contrast, we speculated that low nitrogen availability is linked to the development of colour because betacyanin (which provides the red colour to beetroot) has two atoms of nitrogen in its chemical structure [19]. Compared to the white root ends of beetroot reported by Weaver and Bruner [26], the dispersed white colour in the root systems found in our experiment presents a challenge in assessing the age of roots in specific root segments based on morphological traits. However, the strong link between white colour and average root age indicated that white roots (on a specific date or specific soil layer) could be used as a proxy of root vigour when investigating root function linked to eco-physiology. Detailed studies are needed to elucidate the mechanisms driving the occurrence of dispersed white roots in beetroot.

### Underestimation of beetroot roots due to the presence of white roots

Identification of beetroot roots based on the colour detected in mono-cropped beetroot caused the beetroot roots to be underestimated in the soil layers in August and October. For instance, coloured roots were underestimated by 1% to 22% in August and 7% to 22% roots in October (Fig. 2), confirming our second hypothesis. The largest underestimation was detected in the deepest soil layer in both August (1 − 1.5 m) and October (1.5 − 2 m), due to higher white root counts. This underestimation indicated a systematic, but not constant, error when root proliferation rates were high, and roots were of young age. Tracing white roots backwards and forwards to coloured parts reduced the extent to which beetroot roots were underestimated to 0.5–15% in the soil profile. Thus, it is important to trace white roots backwards and forwards to improve the accuracy of root identification when beetroot is used as a model crop for studying root competition in intercropping systems. There is no evidence that dispersed white roots have been addressed in previous studies using the red colour of beetroot roots (e.g., Andersen et al. [2].

The systematic error in root identification based on root colour and traceability is highlighted here, with this study confirming that this issue causes the root distribution of beetroot to be underestimated, and that of companion crops to be overestimated, in intercropping or multispecies cropping systems. Therefore, root identification based on both root colour and traceability must be improved before using it as a standard to evaluate other new techniques for root identification [8]. For instance, other techniques based on the chemical composition of roots, such as infrared spectroscopy [14], could be combined with the rhizotrons or minirhizotron methods to support root identification [15].

### The traceability of white roots is not influenced by the intercropping system

The traceability of white roots backwards or forwards to red-coloured roots (i.e., the proportion of WC to C counts in our study) was not influenced by the cropping system (mono- versus intercrop), even though C count increased. This result contradicted our fourth hypothesis that intercropping influences the occurrence of white roots compared to monocropping, as shown by white roots identified as beetroot roots by tracing them back to the red parts of the roots. Since the white roots of beetroot could not be differentiated from white cabbage roots, it was not possible to directly confirm how intercropping affects the white roots (W) of beetroot. Therefore, we used white roots that could be traced back to red-coloured roots as a proxy examining the effect of intercropping on the root colour of beetroot. The root systems of species can be influenced by intercropping via competition, recognition, or root N foraging strategies [20, 27, 28]. The increase in WC counts could be the result of beetroot root growth being stimulated by intercropping or regulating pigment biosynthesis through interspecific interactions [9], or both. A similar proportion of WC to C counts between the two cropping systems indicated that intercropping did not influence the colour of small root segments. This finding supports the continued use of beetroot as a model crop in intercropping studies, as long as the root counts of beetroots in intercropping systems are validated by using white root/red root proportions in corresponding soil layers in monocropping systems. Roots of companion crops can subsequently be calculated by subtracting white root count of beetroot from total white root count.

## Conclusions

The abundance of the white roots of beetroot exhibited a spatiotemporal pattern, increasing over time and with soil depth. The proportion of white root count to T count in the deeper soil layer reached 6.9% in August (1 − 1.5 m) and 11.6% in October (1.5 − 2 m). The high abundance of white roots in these deep soil layers indicated that root colour and average root age are linked in given soil layers. When beetroot was used as a model crop for intercropping study and root colour was used as the criterion for root identification, the dispersed white roots of beetroot could cause beetroot roots to be underestimated by 1–22%. Tracing white roots backwards and forwards is recommended during root registration, which could decrease the underestimates to 0.5–15%. Intercropping did not influence the traceability of white roots, therefore root count of beetroot can be validated by a corresponding beetroot in monocropping system. Future studies on the root colour of beetroot and the effects of intercropping on root colour are needed to increase the accuracy of root discrimination and to reveal the potential of using beetroot for studying root age.

### Supplementary Information

**Additional file 1:** **Figure S1.** The dispersed white coloured roots among red roots of beetroot, cultivar Forono (a and b) in observation windows of minirhizotrons in the field experiment under the mono-cropped system; examples from 1 (#29) and 1.15 (#33) meters depth; and cultivars Forono (c), Kogel-2 (d) and Cylindra (e) in cut windows of the pot in the pot trial. **Figure S2.** Registered white roots (green circle) were coloured in a length fraction (red circle) that was positioned outside of the observation window (a) or inside the previous observation window (b) in the field experiment under the mono-cropped system (cultivar Forona). **Figure S3.** Dispersed white roots found in observation windows of minirhizotrons (red arrows) in the field experiment under the mono-cropped system (cultivar Forona). **Table S1.** The proportions of white colored roots, red roots and other colored roots of beetroot to total roots in the pot trial by visual observation after root extraction from soil (*n*=3). Data shown are mean ± s.e.

## Data Availability

All data generated or analysed during this study are included in this published article (and its supplementary information files). All the data can be available by contacting the corresponding author at a reasonable request.

## Abbreviations

VC: Visually coloured root
VW: Visually white root
WC: Traceable white root to coloured root
C: Coloured root
W: White root
T: Total roots
