# Supplementary material for: Dispersed white roots in red beetroot influence the accuracy of root identification based on colours for intercropping studies
Source: BMC Plant Biol. 2023 Sep 9;23:416. doi: 10.1186/s12870-023-04414-5 (PMC10492384; doi:10.1186/s12870-023-04414-5)
Supplement: Supplementary file 1 — Additional file 1: Figure S1. The dispersed white coloured roots among red roots of beetroot, cultivar Forono (a and b) in observation windows of minirhizotrons in the field experiment under the mono-cropped system; examples from 1 (#29) and 1.15 (#33) meters depth; and cultivars Forono (c), Kogel-2 (d) and Cylindra (e) in cut windows of the pot in the pot trial. Figure S2. Registered white roots (green circle) were coloured in a length fraction (red circle) that was positioned outside of the observation window (a) or inside the previous observation window (b) in the field experiment under the mono-cropped system (cultivar Forona). Figure S3. Dispersed white roots found in observation windows of minirhizotrons (red arrows) in the field experiment under the mono-cropped system (cultivar Forona). Table S1. The proportions of white colored roots, red roots and other colored roots of beetroot to total roots in the pot trial by visual observation after root extraction from soil (n=3). Data shown are mean ± s.e. [file 12870_2023_4414_MOESM1_ESM.docx]

**Supplementary Material for**

**Dispersed white roots in red beetroot influence the accuracy of root identification based on colours for intercropping study**

**Journal: BMC Plant Biology**

**Yue Xie^a,b^, Sindhuja Shanmugam^a^, Hanne Lakkenborg Kristensen^a^***

^a^ Department of Food Science, Aarhus University, 8200 Aarhus N, Denmark

^b^ Department of Vegetables, College of Horticulture, China Agricultural University, Beijing, 100193, China

*Corresponding author: [hanne.kristensen@food.au.dk](mailto:hanne.kristensen@food.au.dk)

Department of Food Science, Aarhus University, Agro Food Park 48, 8200 Aarhus N, Denmark.

Figure S1 The dispersed white coloured roots among red roots of beetroot, cultivar Forono (a and b) in observation windows of minirhizotrons in the field experiment under the mono-cropped system; examples from 1 (#29) and 1.15 (#33) meters depth; and cultivars Forono (c), Kogel-2 (d) and Cylindra (e) in cut windows of the pot in the pot trial.


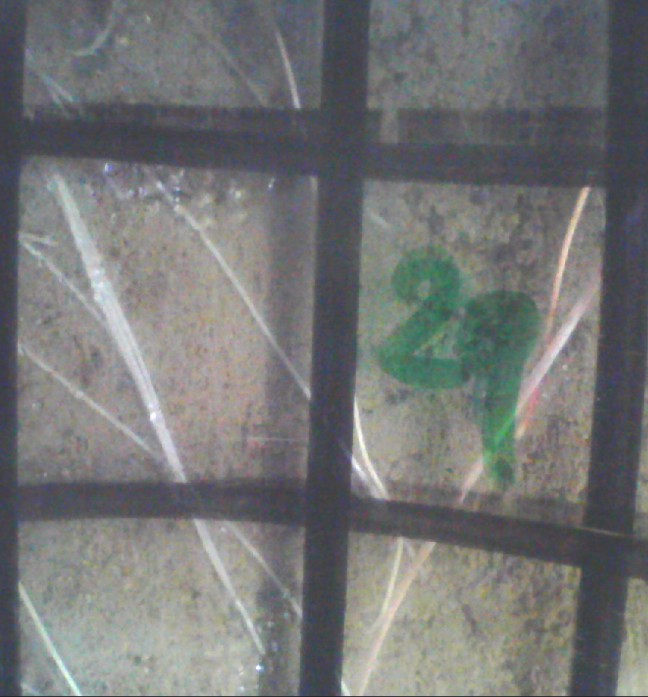


**a**


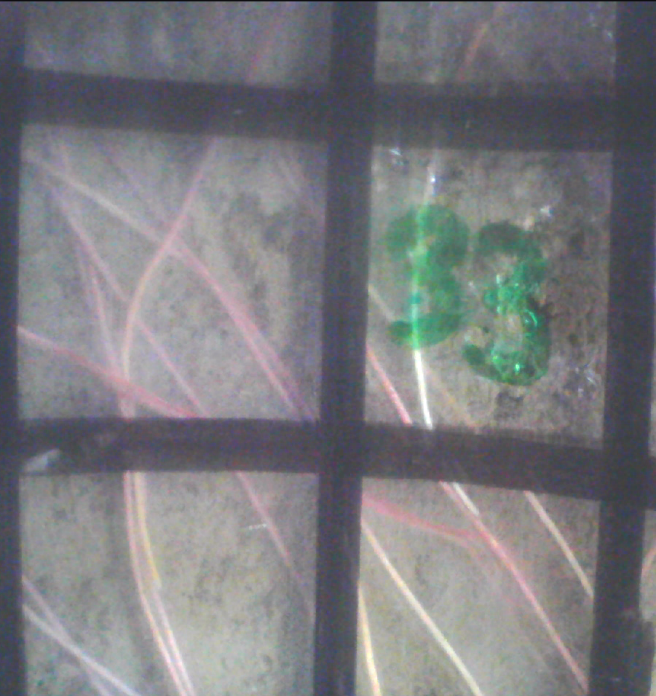


**b**


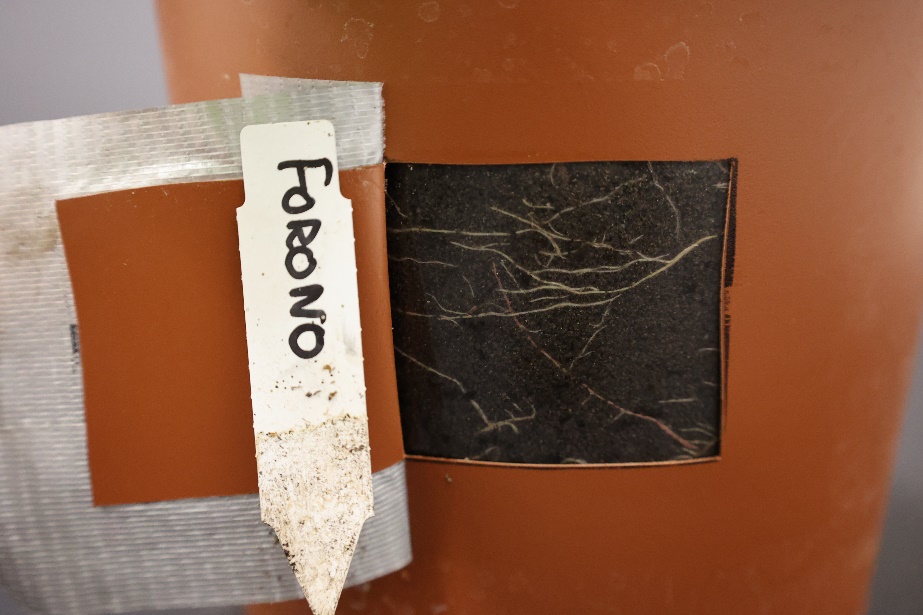


**c**


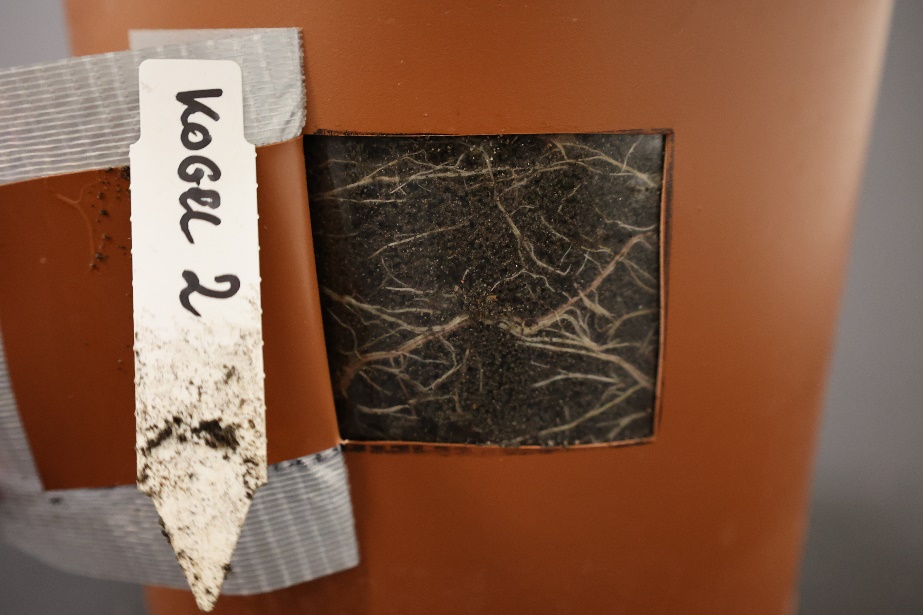


**d**


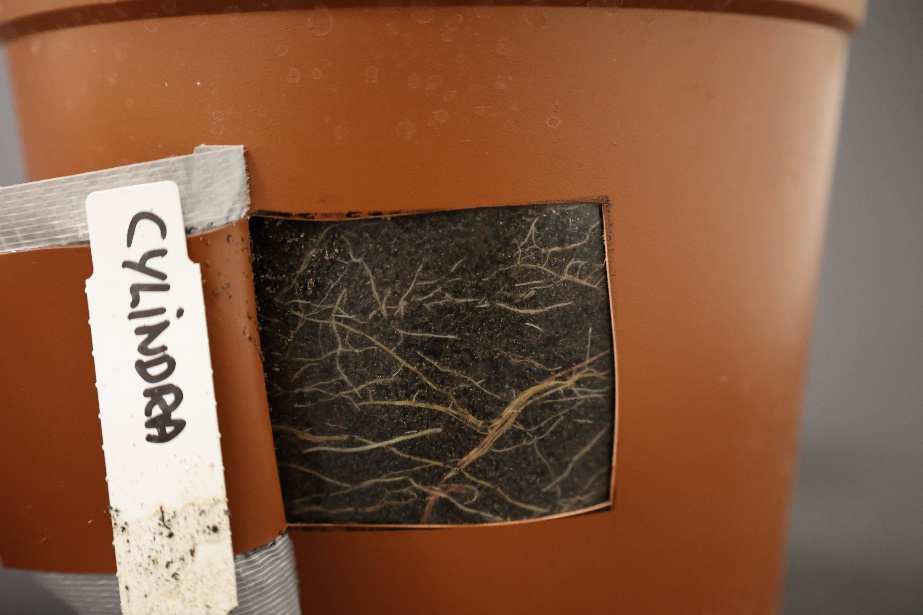


**e**

Figure S2. Registered white roots (green circle) were coloured in a length fraction (red circle) that was positioned outside of the observation window (a) or inside the previous observation window (b) in the field experiment under the mono-cropped system (cultivar Forona).


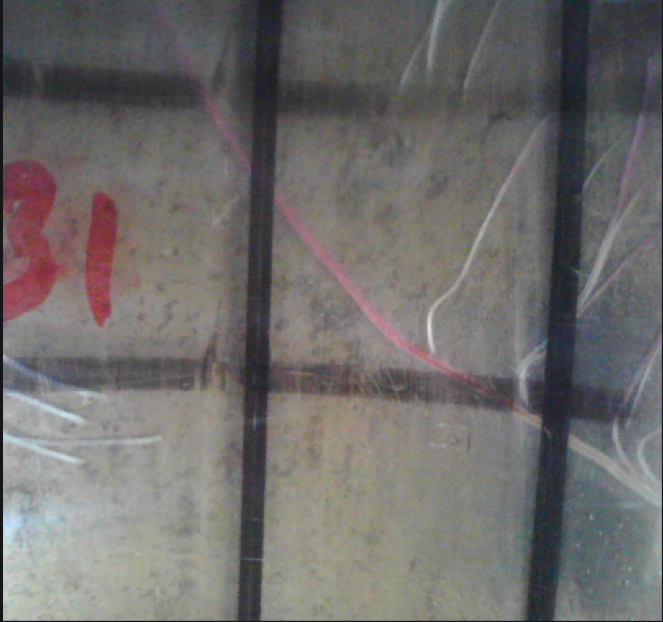

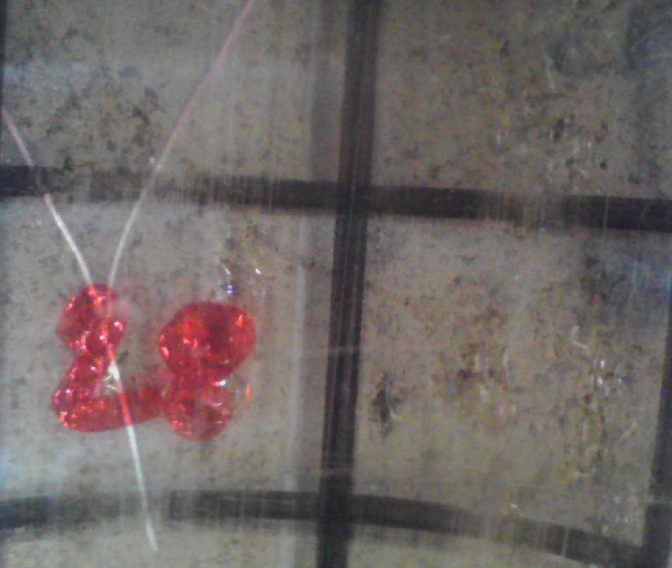


**a**

**b**

Figure S3. Dispersed white roots found in observation windows of minirhizotrons (red arrows) in the field experiment under the mono-cropped system (cultivar Forona).


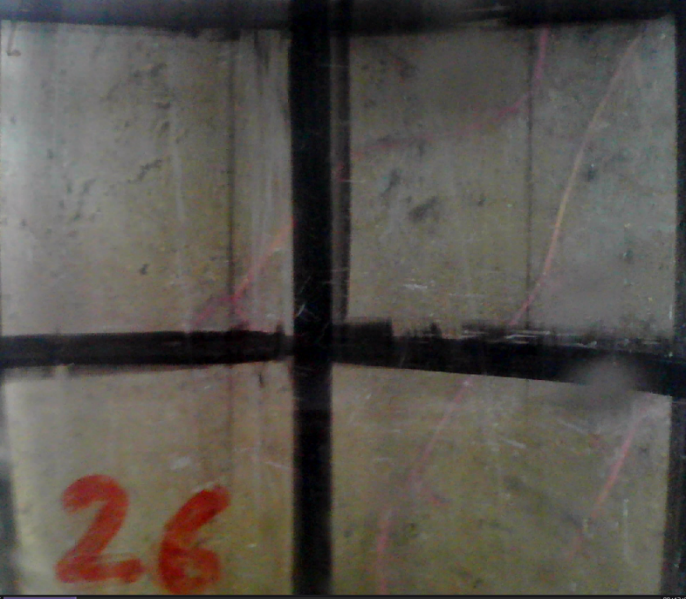

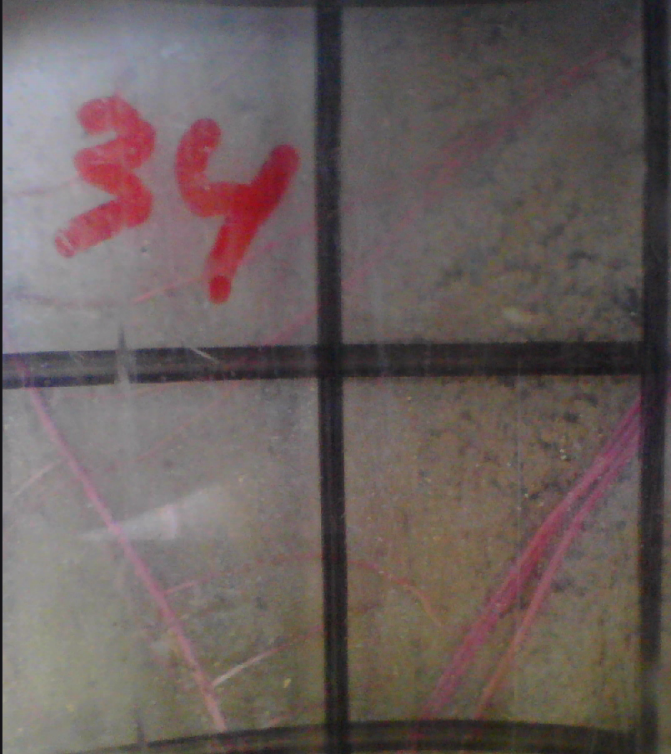

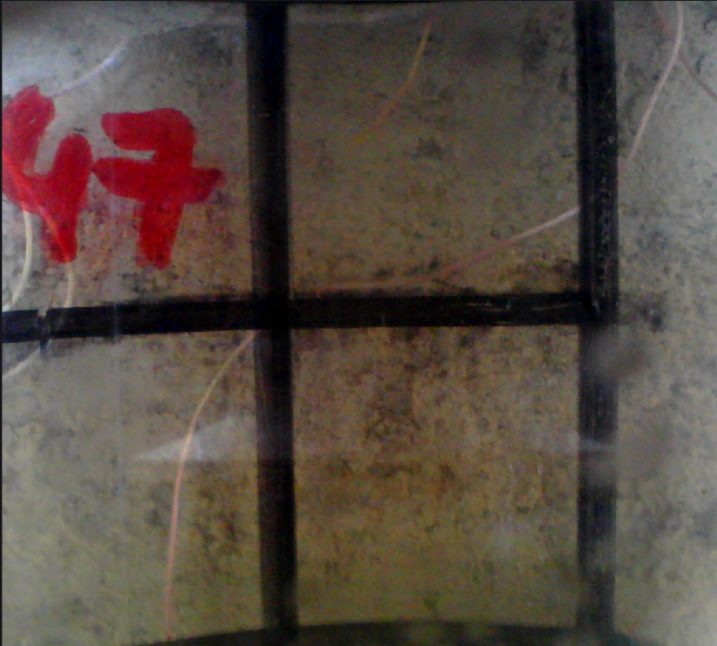

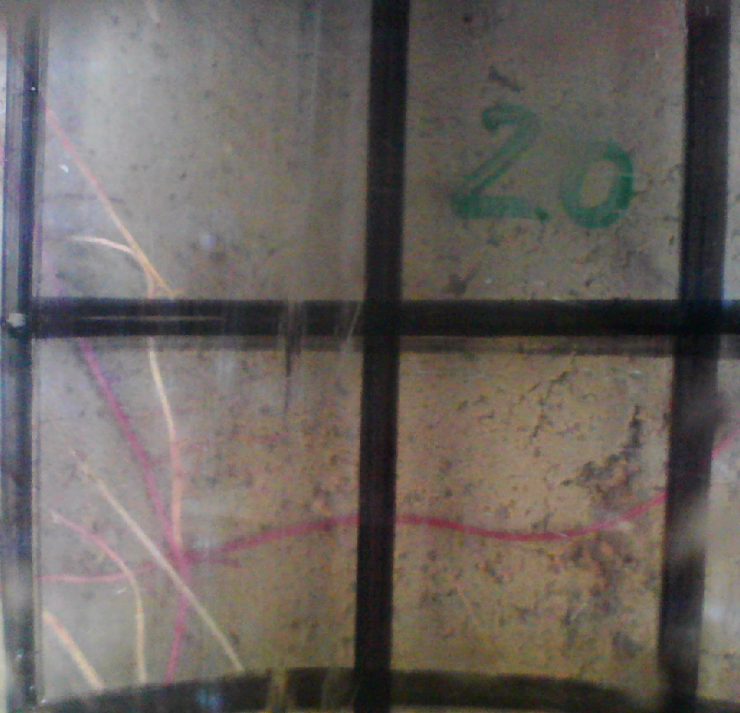


Table S1 The proportions of white colored roots, red roots and other colored roots of beetroot to total roots in the pot trial by visual observation after root extraction from soil (n=3). Data shown are mean ± s.e.

| Cultivar | White roots (%) | Red roots (%) | Other colored roots (%) |
| --- | --- | --- | --- |
| Forona | 13 ± 4 | 39 ± 6 | 48 ± 6 |
| Kogel-2 | 24 ± 4 | 35 ± 5 | 42 ± 5 |
| Cylindra | 3 ± 1 | 62 ± 5 | 35 ± 5 |
